# Reliability of modified sensory interaction test as measured with force platform

**DOI:** 10.1007/s11517-015-1259-x

**Published:** 2015-03-07

**Authors:** Darja Rugelj, Ajda Hrastnik, France Sevšek, Renata Vauhnik

**Affiliations:** Biomechanical Laboratory, Faculty of Health Sciences, University of Ljubljana, Zdravstvena pot 5, 1000 Ljubljana, Slovenia

**Keywords:** Stabilometry, Test–retest reliability, Balance, Sensory conditions, Young, Elderly, Women

## Abstract

The test–retest reliability of the modified sensory interaction test on a force platform was performed in a group of 26 young and 15 elderly females for four sensory conditions: standing on firm and compliant surface with eyes open and closed. The test–retest reliability was good to excellent in both groups, with higher level of test–retest reliability in more demanding conditions. The most reliable time-domain variables for standing on firm surface with eyes open were: sway area from principal components (ICC = 0.77) for young and mean velocity, medio-lateral and total path lengths (ICC = 0.91) for elderly. For eyes closed, the most reliable variables were antero-posterior path length and sway area calculated by Fourier coefficients (ICC = 0.85) for young and medio-lateral path length (ICC = 0.93) for elderly. For compliant surface with open eyes, the most reliable variable was medio-lateral variability (ICC = 0.83) for young and total path length and mean velocity (ICC = 0.92) for elderly participants, whereas for eyes closed the most reliable variables were mean velocity, total and medio-lateral path lengths for young, and mean velocity for elderly group, all with ICC = 0.90. Modified sensory interaction test is therefore a reliable measure for balance and could be recommended as an outcome measure for balance retraining programmes.

## Introduction

Postural sway during unperturbed stance is a consequence of constant adjustments of body segments. The amount of sway depends on the quality and quantity of the afferent sensory flow as well as on age or different diseases. Body sway motion is primarily detected by the visual, vestibular and proprioceptive sensory systems. Information from all sensory systems is not always available or accurate, and therefore, the postural control system must adjust to maintain steady stance in various environmental conditions [19]. Several tests are addressing the contribution of individual sensory information and a combination of sensory information to the ability of the maintenance of steady posture. The classical Romberg test (standing with eyes closed) has been upgraded into a sensory interaction test (SIT) [34] and has been used clinically since then. Modified sensory interaction test (mSIT) measures balance during unsupported standing in four different sensory conditions: standing on a firm surface and on a compliant surface with eyes open and closed. Measurement of human body centre of pressure (CoP) movement (body or postural sway) with a force platform is a standard procedure for the assessment of postural stability. Rugelj et al. [26] and Domijan et al. [21] performed the mSIT on a force platform that allowed monitoring of various variables related to the CoP movements. In those two studies, the mSIT on a force platform was used as an outcome measure test. However, the reliability of mSIT as a test performed for all four test conditions on a force platform has been tested only in a group of young gymnasts with various musculoskeletal problems [11].

Despite the fact that the reliability of force platform measurements has been intensively evaluated [8, 11, 14, 20, 23, 28, 29], the results are still inconclusive and difficult to compare. Different foot positions and time for data acquisition, which all affect the reliability, were studied by the researchers. Followed by an initiative of the ISPGR (Bologna, Italy 2009), standardisation of clinical stabilometry was proposed by Scoppa et al. [31] according to which for stable values of different CoP variables, a minimum of 30-s acquisition time is required and sampling frequency of at least 50 Hz is suggested. All the here-referenced studies used 60-s time for the data acquisition and time-domain parameters. The reliability studies of fractal dimensions are even more inconclusive as the authors use different calculation procedures: from the box-counting method [6], to the circular and ellipsoidal perimeter evaluation of the two-dimensional CoP trajectory [22, 29], and the fractal dimension calculation of the antero-posterior and medio-lateral CoP time series [7, 12].

For the traditional time-domain variables in a group of young participants standing on a firm surface with open eyes, Santos et al. [29] reported poor to moderate reliability (ICC = 0.53–0.76). With eyes closed, the reported reliability was also poor to moderate (ICC = 0.02–0.72). On the other hand, Pinsault et al. [20] and Lin et al. [14] also in a group of young persons reported good to excellent reliability (ICC ranging from 0.77 to 0.94). In elderly population, a good test–retest reliability has been reported for measurements during standing on a firm surface with eyes open (ICC = 0.51–0.89) [36] and closed (ICC = 0.64–0.89 [36] and ICC = 0.57–0.92 [14]). For fractal dimensions, Doyle et al. [7] reported fair to excellent test–retest reliability (ICC = 0.62–0.90) using Higutchi algorithm [12] and only 10-s measuring time, while Santos et al. [29] reported the fractal dimension reliability to be comparable to the other measures for 60-s measurement time and using circular and ellipsoidal perimeter evaluation of the two-dimensional CoP trajectory.

For standing on compliant surface, Harringe et al. [11] reported poor reliability in a group of young injured gymnasts standing on compliant surface with eyes open (ICC = 0.50) and standing on compliant surface with eyes closed (ICC = 0.51). The study of Salavati et al. [28] reported higher reliability on a foam surface with eyes closed (ICC = 0.74) in a group of persons with low back pain, ACL injury and ankle sprain. Further, a good test–retest has been reported for standing on a foam surface with eyes closed for a group of elderly participants (ICC ranged from 0.65 to 0.85) [15].

The available test–retest reliability studies of mSIT for all four sensory conditions are by Harringe et al. [11], followed by Salavati et al. [28] and Moghadam et al. [15] for three sensory conditions. Therefore, the purpose of the present study was to assess the test–retest reliability of the mSIT for four measurement conditions on a force platform in a group of young and elderly healthy women. Furthermore, the most reliable CoP variables across the four sensory conditions were to be identified. For this purpose, eight variables in time domain and four fractal dimensions were considered. Time-domain variables were antero-posterior and medio-lateral variability (variability calculated as the standard deviation of the position of the CoP on the force platform), mean velocity, medio-lateral and antero-posterior path lengths, total CoP path length, sway area as determined by principal component analysis (PCA) [16] and sway area as determined by Fourier coefficients [25] for the time domain. Additionally, the fractal dimensions of the medio-lateral and antero-posterior time series were determined for short and long time intervals.

## Methods

### Subjects

Twenty-six young (21.4 ± 2.5 years) and 15 elderly (73.2 ± 6.5 years) women participated in the study. Their anthropometric data are presented in Table 1. The inclusion criteria were female gender and no prior injuries or other conditions which could affect their balance. Their Romberg quotients indicated good proprioceptive function (Table 1). The study was approved by Medical Ethic Committee, and prior to any measurements, all participants read information about the test protocol, received additional verbal explanations when required and provided written informed consent.

**Table 1:** Anthropometric data of the young and elderly women and Romberg quotient (RQ) for two postural sway variables

Body characteristics	Young	Elderly
Age (years)	21.4 ± 2.5	73.2 ± 6.5
Body mass (kg)	63.7 ± 14.8	68.73 ± 13.6
Body height (cm)	167.0 ± 6.3	163.6 ± 5.49
RQ total path length	1.47 ± 0.24	1.44 ± 0.26
RQ sway area PCA	1.50 ± 0.66	1.20 ± 0.45

### Instrumentation

The force platform Kistler 9286AA (Winterthur, Switzerland) with the corresponding data acquisition software BioWare was used to assess the CoP movement during upright quiet standing. The system was calibrated by the producer and periodically checked using static loads. For the compliant surface measurements, an Airex mat (40 × 48 × 6 cm) was positioned on the force platform with a non-slip rubber pad in-between. The raw data of 60-s measurements were collected and stored on disk of a PC-type computer with Kistler’s BioWare program using 50-Hz sampling rate. All the analyses were later performed on a Linux server (Fedora 18) with a specially developed software. This is a web-based application that had been developed for our stabilometric measurements and consists of system procedures and data analysis programs written in C, Fortran and PHP, while the graph plotting is done with the open-code program Gnuplot. This application is publically accessible [32].

**Fig. 1:**
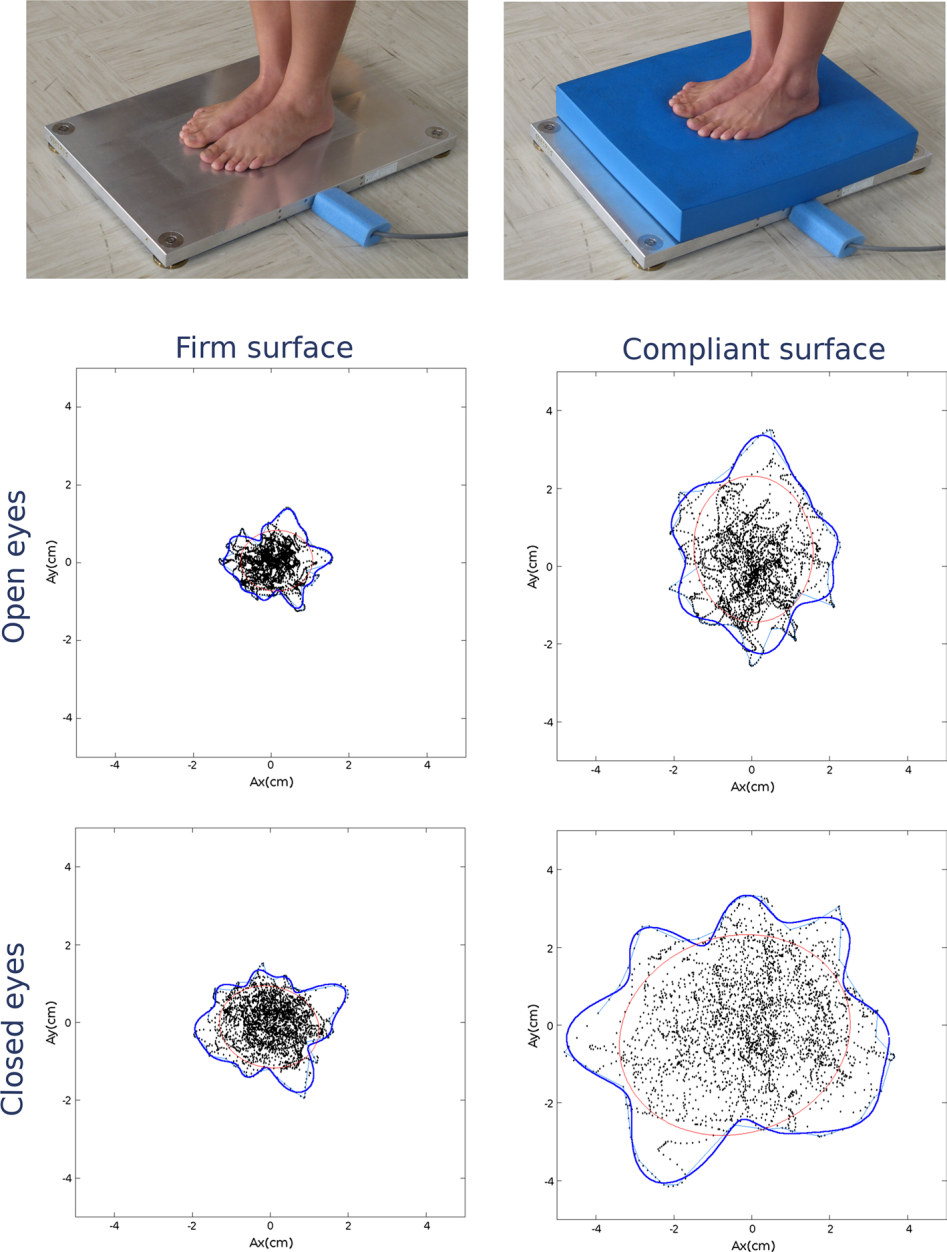
Measuring procedure on a *firm* and *compliant* surface and typical stabilograms for an elderly person. The experimental data are represented by *dots*; the outline (*blue*) was calculated using 100 angular intervals and 20 Fourier coefficients, whereas the ellipse (*red*) results from the principal component analysis

To exclude the possible effect of initial posture adjustments, participants stood on the platform for 5 to 10 s prior to data acquisition. Standard statistical parameters were calculated, such as standard deviations and the averages of the absolute values of the CoP displacements and velocities, as well as the total path length and the lengths of the medio-lateral and antero-posterior CoP movements as well as the frequency distributions and the fractal dimensions [25]. The outline of the CoP movement area has been determined by considering the extreme points in 100 angular intervals to which an analytical expression was fitted [33]. This expression determines the distance from the chosen centre at the angle* φ* as the sum of *m*_max_ terms of the type: (*A*_*m*_ cos *mφ* + *B*_*m*_ sin *mφ*), where *A*_*m*_ and *B*_*m*_ are the Fourier coefficients to be fitted and *m*_max_ the maximal number of the coefficients used. With 100 angular intervals for the outline calculation, the value of *m*_max_ = 20 was found to be appropriate. From thus calculated Fourier coefficients, the shape of the postural sway area was determined and its area calculated [25]. The stabilogram area was also calculated using principal component analysis (PCA) [16] where the area is represented by an ellipse with the principal axes determined from the eigenvalues (*s*_0_) of the covariant matrix as 1.96 *s*_0_ (Fig. 1).

The fractal dimensions of the medio-lateral and antero-posterior CoP position time series were determined by the Higutchi method [12]. The average curve lengths were calculated as a function of time interval, and the fractal dimension was then obtained by linear regression from the slope of the log/log graph [27]. As these graphs indicated two different slopes for the short and long time intervals, they were analysed separately. The time intervals were chosen to be from 0 to 0.3 s (short) and from 0.8 to 12 s (long) to exclude the intermediate transition region.

Eight sway parameters in time domain were chosen for this analysis: (1) variability of the CoP position in the medio-lateral and (2) antero-posterior directions calculated as the standard deviations of the medio-lateral and antero-posterior variability of the position of CoP on the force platform, (3) mean of the absolute value of the CoP velocity during the 60-s data acquisition interval, (4) medio-lateral and (5) antero-posterior path lengths, (6) total path length, (7) sway area calculated by PCA, (8) sway area determined as the best area outline represented by the first 20 Fourier coefficients (FAO) as described above. Additionally, four fractal dimensions were chosen for this analysis: medio-lateral and antero-posterior time series dimensions for short and long time intervals. Thus, altogether twelve sway parameters were analysed.

### Procedure

In order to assess the test–retest reliability of mSIT, a set of four measurements were performed, each with a different sensory condition. They were performed twice, 1 week apart. These were as follows: (1) standing on a firm surface with eyes open (FO), (2) standing on a firm surface with eyes closed (FC), (3) standing on a compliant surface with eyes open (CO), (4) standing on a compliant surface with eyes closed (CC) (Fig. 1).

Each test lasted 60 s. Participants were instructed to stand barefooted with feet close together on the force platform, with or without the Airex mat, as required by the measurement. Subjects were asked to stand as still as possible. Arms were relaxed beside the body. Head was held upright with eyes facing straight forward to the point marked on a board 2 m away at the eye height. Depending on the measurement being performed, the subjects had their eyes open or closed. Measuring procedure was immediately interrupted, if the subject opened eyes, moved feet or arms from the required position or even stepped off the force platform. Between the measurements, participants were asked to sit down for 60 s to have a rest.

### Statistical methods

The Statistical Package for Social Sciences (SPSS 20, SPSS Inc., Chicago, IL, USA) was used for statistical analysis. To determine the test–retest reliability, intraclass correlation coefficients (ICC 2,1) with 95 % confidence intervals (CI 95 %) and the standard errors of measurement (SEM) were calculated [1].

## Results

### Test–retest reliability of time-domain variables

#### Standing on a firm surface with eyes open

In the group of young women, the values of ICC ranged from 0.56 to 0.77, where medio-lateral variability was the least reliable parameter, and sway area as calculated with PCA was the most reliable one. In the group of elderly women, ICC ranged from 0.80 to 0.91, where medio-lateral variability was the least reliable parameter, and mean velocity, total path length and medio-lateral path length were the most reliable ones. The data for these variables are presented in Table 2.

**Table 2:** Standing on a firm surface with eyes open: values of individual variables for the first and second measurements, their absolute difference with intraclass correlation coefficients (ICC) with bottom and top limits of 95 % confidence intervals (CI)

	Age group	1st measurement Average ± SD (SEM)	2nd measurement Average ± SD (SEM)	ICC	95 % CI
Medio-lateral variability (cm)	Young	0.44 ± 0.1 (0.02)	0.47 ± 0.13 (0.03)	0.56	0.04–0.80
	Elderly	0.57 ± 0.08 (0.02)	0.56 ± 0.13 (0.03)	0.80	0.41–0.93
Antero-posterior variability (cm)	Young	0.51 ± 0.16 (0.03)	0.49 ± 0.18 (0.04)	0.62	0.15–0.83
	Elderly	0.62 ± 0.19 (0.05)	0.61 ± 0.21 (0.06)	0.87	0.63–0.96
Medio-lateral path length (cm)	Young	51. 88 ± 8.78 (1.72)	48.76 ± 10.22 (2.00)	0.68	0.31–0.86
	Elderly	80. 16 ± 17.55 (4.53)	75.93 ± 21.69 (5.60)	0.91	0.73–0.97
Antero-posterior path length (cm)	Young	46.54 ± 9.28 (1.82)	43.83 ± 10.48 (2.06)	0.65	0.24–0.84
	Elderly	61.14 ± 15.67 (4.05)	60.52 ± 15.24 (3.94)	0.85	0.56–0.95
Total path length (cm)	Young	77.52 ± 12.85 (2.52)	72.93 ± 15.03 (2.95)	0.69	0.32–0.86
	Elderly	111.60 ± 21.33 (5.51)	107.85 ± 26.43 (6.82)	0.91	0.73–0.97
Mean velocity (cm/s)	Young	1.29 ± 0.21 (0.04)	1.22 ± 0.25 (0.05)	0.69	0.32–0.86
	Elderly	1.86 ± 0.36 (0.09)	1.80 ± 0.44 (0.11)	0.91	0.73–0.97
Sway area FAO (cm^2^)	Young	3.56 ± 1.38 (0.28)	3.67 ± 1.79 (0.35)	0.74	0.42–0.89
	Elderly	6.32 ± 2.04 (0.53)	6.50 ± 3.00 (0.78)	0.88	0.65–0.96
Sway area PCA (cm^2^)	Young	2.61 ± 1.19 (0.23)	2.81 ± 1.61 (0.32)	0.77	0.49–0.90
	Elderly	4.11 ± 1.45 (0.38)	4.09 ± 1.71 (0.44)	0.85	0.56–0.95
Fractal dimension ML short interval	Young	1.10 ± 0.02 (0.00)	1.10 ± 0.02 (0.00)	0.67	0.27–0.85
	Elderly	1.09 ± 0.02 (0.01)	1.09 ± 0.02 (0.01)	0.90	0.70–0.97
Fractal dimension ML long interval	Young	1.81 ± 0.13 (0.02)	1.77 ± 0.13 (0.03)	0.57	0.05–0.81
	Elderly	1.85 ± 0.11 (0.03)	1.88 ± 0.10 (0.03)	0.56	−0.31–0.85
Fractal dimension AP short interval	Young	1.18 ± 0.04 (0.01)	1.17 ± 0.04 (0.01)	0.85	0.66–0.93
	Elderly	1.13 ± 0.03 (0.01)	1.11 ± 0.03 (0.01)	0.88	0.64–0.96
Fractal dimension AP long interval	Young	1.67 ± 0.14 (0.03)	1.68 ± 0.16 (0.03)	0.34	−0.48–0.70
	Elderly	1.76 ± 0.14 (0.04)	1.77 ± 0.15 (0.04)	0.77	0.30–0.92

*SD* standard deviation, *ML* medio-lateral, *AP* antero-posterior, *FAO* Fourier analysis outline, *PCA* principal component analysis, short interval 0–0.3 s, long interval 0.8–12 s

#### Standing on a firm surface with eyes closed

In the group of young women, the values of ICC ranged from 0.40 to 0.85, where the medio-lateral variability, expressed as the average position of CoP, was the least reliable parameter and the most reliable parameters were antero-posterior path length of CoP and the sway area as calculated by Fourier coefficients (FAO). In the group of elderly women, the values of ICC ranged from 0.61 to 0.93, where the sway area as calculated by Fourier coefficients (FAO) was least reliable parameter and the most reliable parameter was medio-lateral path length of CoP. The data for these variables are presented in Table 3.

**Table 3:** Standing on a firm surface with eyes closed: values of individual variables for the first and second measurements, their absolute difference with intraclass correlation coefficients (ICC) with bottom and top limits of 95 % confidence intervals (CI)

	Age group	1st measurement Average ± SD (SEM)	2nd measurement Average ± SD (SEM)	ICC	95 % CI
Medio-lateral variability (cm)	Young	0.53 ± 0.14 (0.03)	0.55 ± 0.14 (0.03)	0.40	−0.35–0.73
	Elderly	0.62 ± 0.14 (0.04)	0.64 ± 0.11 (0.03)	0.89	0.68–0.96
Antero-posterior variability (cm)	Young	0.56 ± 0.25 (0.05)	0.51 ± 0.16 (0.03)	0.82	0.60–0.92
	Elderly	0.62 ± 0.16 (0.04)	0.66 ± 0.19 (0.05)	0.65	−0.04–0.88
Medio-lateral path length (cm)	Young	77.21 ± 21.14 (4.23)	70.86 ± 17.20 (3.44)	0.77	0.48–0.90
	Elderly	114.99 ± 29.89 (7.72)	115.32 ± 26.36 (6.81)	0.93	0.78–0.98
Antero-posterior path length (cm)	Young	69.80 ± 20.18 (4.04)	64.25 ± 15.38 (3.08)	0.85	0.67–0.94
	Elderly	87.78 ± 27.42 (7.08)	86.71 ± 18.89 (4.88)	0.85	0.55–0.95
Total path length (cm)	Young	115.68 ± 30.87 (6.17)	106.35 ± 24.12 (4.82)	0.83	0.61–0.93
	Elderly	160.35 ± 39.56 (10.22)	159.78 ± 31.89 (8.23)	0.89	0.68–0.96
Mean velocity (cm/s)	Young	1.93 ± 0.52 (0.10)	1.77 ± 0.40 (0.08)	0.83	0.61–0.93
	Elderly	2.67 ± 0.66 (0.17)	2.66 ± 0.53 (0.14)	0.89	0.68–0.96
Sway area FAO (cm^2^)	Young	5.64 ± 3.26 (0.65)	5.43 ± 2.64 (0.53)	0.85	0.66–0.93
	Elderly	8.28 ± 3.31 (0.85)	9.40 ± 3.30 (0.85)	0.61	−0.17–0.87
Sway area PCA (cm^2^)	Young	3.68 ± 2.52 (0.50)	3.46 ± 1,92 (0.38)	0.84	0.65–0.93
	Elderly	4.72 ± 2.12 (0.55)	5.19 ± 2.02 (0.52)	0.71	0.13–0.90
Fractal dimension ML short interval	Young	1.10 ± 0.02 (0.00)	1.10 ± 0.02 (0.00)	0.80	0.56–0.91
	Elderly	1.11 ± 0.03 (0.01)	1.11 ± 0.01 (0.00)	0.58	−0.27–0.86
Fractal dimension ML long interval	Young	1.93 ± 0.11 (0.02)	1.87 ± 0.08 (0.02)	0.47	−0.19–0.76
	Elderly	1.95 ± 0.05 (0.01)	1.97 ± 0.04 (0.01)	0.65	−0.04–0.88
Fractal dimension AP short interval	Young	1.15 ± 0.04 (0.01)	1.14 ± 0.03 (0.01)	0.74	0.43–0.89
	Elderly	1.14 ± 0.04 (0.01)	1.13 ± 0.04 (0.01)	0.97	0.92–0.99
Fractal dimension AP long interval	Young	1.88 ± 0.09 (0.02)	1.87 ± 0.08 (0.02)	0.39	−0.36–0.73
	Elderly	1.87 ± 0.11 (0.03)	1.90 ± 0.10 (0.03)	0.59	−0.21–0.86

*SD* standard deviation, *ML* medio-lateral, *AP* antero-posterior, *FAO* Fourier analysis outline, *PCA* principal component analysis, short interval 0–0.3 s, long interval 0.8–12 s

#### Standing on a compliant surface with eyes open

In the group of young women, the values of ICC ranged from 0.66 to 0.83. The least reliable parameter was antero-posterior variability, and the most reliable parameter was medio-lateral variability. In the group of elderly women, the values of ICC ranged from 0.72 to 0.92. The least reliable parameter was antero-posterior variability, and the most reliable parameters were mean velocity and total path length. The data for these variables are presented in Table 4.

**Table 4:** Standing on a compliant surface with eyes open: values of individual variables for the first and second measurements, their absolute difference with intraclass correlation coefficients (ICC) with bottom and top limits of 95 % confidence intervals (CI)

	Age group	1st measurement Average ± SD (SEM)	2nd measurement Average ± SD (SEM)	ICC	95 % CI
Medio-lateral variability (cm)	Young	0.65 ± 0.12 (0.02)	0.65 ± 0.09 (0.02)	0.83	0.62–0.92
	Elderly	0.92 ± 0.16 (0.04)	0.89 ± 0.23 (0.06)	0.83	0.50–0.94
Antero-posterior variability (cm)	Young	0.78 ± 0.21 (0.04)	0.81 ± 0.17 (0.03)	0.66	0.23–0.85
	Elderly	0.97 ± 0.22 (0.06)	1.00 ± 0.25 (0.06)	0.72	0.17–0.91
Medio-lateral path length (cm)	Young	96.87 ± 17.97 (3.6)	88.89 ± 16.47 (3.29)	0.77	0.40–0.90
	Elderly	167 ± 34.61 (8.94)	153.70 ± 40.97 (10.58)	0.90	0.69–0.97
Antero-posterior path length (cm)	Young	87.09 ± 15.36 (3.07)	78.82 ± 13.32 (2.66)	0.70	0.20–0.88
	Elderly	142.12 ± 29.25 (7.55)	131.73 ± 37.63 (9.72)	0.90	0.70–0.97
Total path length (cm)	Young	144.63 ± 24.49 (4.9)	131.74 ± 21.44 (4.29)	0.74	0.24–0.90
	Elderly	243.57 ± 46.15 (11.92)	224.85 ± 62.46 (16.13)	0.92	0.77–0.98
Mean velocity (cm/s)	Young	2.41 ± 0.41 (0.08)	2.20 ± 0.36 (0.07)	0.74	0.24–0.90
	Elderly	4.06 ± 0.77 (0.20)	3.75 ± 1.04 (0.27)	0.92	0.77–0.98
Sway area FAO (cm^2^)	Young	9.51 ± 3.6 (0.74)	8.95 ± 2.39 (0.49)	0.67	0.25–0.85
	Elderly	19.04 ± 6.64 (1.71)	17.69 ± 7.32 (1.89)	0.89	0.67–0.96
Sway area PCA (cm^2^)	Young	6.24 ± 2.66 (0.53)	6.26 ± 1.96 (0.39)	0.73	0.38–0.88
	Elderly	10.76 ± 3.78 (0.98)	10.75 ± 4.74 (1.22)	0.84	0.53–0.95
Fractal dimension ML short interval	Young	1.10 ± 0.02 (0.00)	1.10 ± 0.02 (0.00)	0.79	0.52–0.90
	Elderly	1.11 ± 0.02 (0.01)	1.12 ± 0.02 (0.01)	0.80	0.40–0.93
Fractal dimension ML long interval	Young	1.90 ± 0.08 (0.02)	1.90 ± 0.09 (0.02)	0.75	0.45–0.89
	Elderly	1.93 ± 0.04 (0.01)	1.93 ± 0.08 (0.02)	0.73	0.20–0.91
Fractal dimension AP short interval	Young	1.12 ± 0.03 (0.01)	1.12 ± 0.03 (0.00)	0.80	0.55–0.91
	Elderly	1.12 ± 0.02 (0.00)	1.11 ± 0.01 (0.00)	0.13	−1.59–0.71
Fractal dimension AP long interval	Young	1.77 ± 0.13 (0.03)	1.70 ± 0.16 (0.03)	0.46	−0.21–0.76
	Elderly	1.86 ± 0.11 (0.03)	1.78 ± 0.11 (0.03)	0.82	0.45–0.94

*SD* standard deviation, *ML* medio-lateral, *AP* antero-posterior, *FAO* Fourier analysis outline, *PCA* principal component analysis, short interval 0–0.3 s, long interval 0.8–12 s

#### Standing on a compliant surface with eyes closed

In the group of young women, the values of ICC ranged from 0.67 to 0.90. The least reliable variable was again the antero-posterior variability, and the following three variables had ICC value 0.90: total path length of the CoP, mean velocity and medio-lateral path length of CoP. In the group of elderly women, the values of ICC ranged from 0.63 to 0.90. The least reliable variable was again the antero-posterior variability, and the following two variables had ICC value 0.90: total path length and mean velocity of CoP. These variables are presented in Table 5.

**Table 5:** Standing on a compliant surface with eyes closed: values of individual variables for the first and second measurements, their absolute difference with intraclass correlation coefficients (ICC) with bottom and top limits of 95 % confidence intervals (CI)

	Age group	1st measurement Average ± SD (SEM)	2nd measurement Average ± SD (SEM)	ICC	95 % CI
Medio-lateral variability (cm)	Young	1.18 ± 0.18 (0.04)	1.16 ± 0.20 (0.04)	0.76	0.46–0.89
	Elderly	1.51 ± 0.20 (0.06)	1.46 ± 0.29 (0.08)	0.80	0.33–0.94
Antero-posterior variability (cm)	Young	1.33 ± 0.17 (0.03)	1.21 ± 0.20 (0.04)	0.67	0.14–0.87
	Elderly	1.54 ± 0.24 (0.07)	1.49 ± 0.19 (0.05)	0.63	−0.21–0.89
Medio-lateral path length (cm)	Young	222.93 ± 56.79 (11.36)	213.27 ± 55.57 (11.12)	0.90	0.77–0.95
	Elderly	328.61 ± 78.34 (21.73)	314.43 ± 78.10 (21.66)	0.88	0.60–0.96
Antero-posterior path length (cm)	Young	231.83 ± 52.32 (10.46)	213.51 ± 59.36 (11.87)	0.88	0.69–0.95
	Elderly	287.80 ± 61.11 (16.95)	292.80 ± 58.35 (16.18)	0.87	0.59–0.96
Total path length (cm)	Young	357.76 ± 82.37 (16.47)	335.63 ± 87.52 (17.50)	0.90	0.76–0.96
	Elderly	485.07 ± 102.25 (28.36)	476.47 ± 104.15 (28.89)	0.90	0.68–0.97
Mean velocity (cm/s)	Young	5.97 ± 1.37 (0.28)	5.60 ± 1.46 (0.29)	0.90	0.76–0.96
	Elderly	8.09 ± 1.71 (0.47)	7.95 ± 1.74 (0.48)	0.90	0.68–0.97
Sway area FAO (cm^2^)	Young	31.45 ± 7.64 (1.53)	30.18 ± 9.92 (1.98)	0.83	0.62–0.92
	Elderly	51.86 ± 15.08 (4.18)	49.71 ± 14.61 (4.05)	0.69	−0.02–0.91
Sway area PCA (cm^2^)	Young	19.14 ± 4.85 (0.97)	17.17 ± 5.17 (1.03)	0.83	0.57–0.93
	Elderly	28.03 ± 6.77 (1.88)	26.32 ± 7.03 (1.95)	0.87	0.57–0.96
Fractal dimension ML short interval	Young	1.11 ± 0.02 (0.00)	1.11 ± 0.02 (0.00)	0.78	0.50–0.90
	Elderly	1.12 ± 0.03 (0.01)	1.11 ± 0.02 (0.01)	0.93	0.77–0.98
Fractal dimension ML long interval	Young	1.98 ± 0.04 (0.01)	1.97 ± 0.04 (0.01)	0.68	0.29–0.86
	Elderly	1.99 ± 0.03 (0.01)	1.99 ± 0.02 (0.01)	0.16	−1.77–0.74
Fractal dimension AP short interval	Young	1.15 ± 0.05 (0.01)	1.14 ± 0.04 (0.01)	0.86	0.70–0.94
	Elderly	1.14 ± 0.03 (0.01)	1.13 ± 0.03 (0.01)	0.85	0.49–0.95
Fractal dimension AP long interval	Young	1.98 ± 0.04 (0.01)	1.98 ± 0.04 (0.01)	0.65	0.22–0.84
	Elderly	1.97 ± 0.04 (0.01)	1.99 ± 0.03 (0.01)	0.33	−1.19–0.80

*SD* standard deviation, *ML* medio-lateral, *AP* antero-posterior, *FAO* Fourier analysis outline, *PCA* principal component analysis, short interval 0–0.3 s, long interval 0.8–12 s

### Test–retest reliability of fractal dimension variables

#### Standing on a firm surface with eyes open

In the group of young women, the values of ICC ranged from 0.34 to 0.85. The least reliable variable was the antero-posterior fractal dimension for long time interval (0.8 to 12 s), whereas the most reliable one was the antero-posterior fractal dimension for short time interval (0 to 0.3 s). In the group of elderly women, the values of ICC ranged from 0.56 to 0.90. The least reliable variable was the medio-lateral fractal dimension for long time interval, whereas the most reliable one was the medio-lateral fractal dimension for short time interval (Table 2).

#### Standing on a firm surface with eyes closed

In the group of young women, the values of ICC ranged from 0.39 to 0.80. The least reliable variable was the antero-posterior fractal dimension for long time interval (0.8 to 12 s), and the most reliable parameter was the medio-lateral fractal dimension for short time interval (0 to 0.3 s). In the group of elderly women, the values of ICC ranged from 0.58 to 0.97. The least reliable variable was the medio-lateral fractal dimension for short time interval, and the most reliable parameter was the antero-posterior fractal dimension for short time interval (Table 3).

#### Standing on a compliant surface with eyes open

In the group of young women, the values of ICC ranged from 0.46 to 0.80 The least reliable variable was the antero-posterior fractal dimension for long time interval (0.8 to 12 s), and the most reliable parameter was the antero-posterior fractal dimension for short time interval (0 to 0.3 s). In the group of elderly women, the values of ICC ranged from 0.13 to 0.82. The least reliable variable was the antero-posterior fractal dimension for short time interval, and the most reliable parameter was the antero-posterior fractal dimension for long time interval (Table 4).

#### Standing on a compliant surface with eyes closed

In the group of young women, the values of ICC ranged from 0.65 to 0.86. The least reliable variable was the antero-posterior fractal dimension for long time interval (0.8 to 12 s), and the most reliable parameter was the antero-posterior fractal dimension for short time interval (0 to 0.3 s). In the group of elderly women, the values of ICC ranged from 0.16 to 0.93. The least reliable variable was the medio-lateral fractal dimension for long time interval, and the most reliable parameter was the medio-lateral fractal dimension for short time interval (Table 5).

## Discussion

The purpose of the study was to determine the test–retest reliability of the mSIT performed on a force platform for twelve sway variables in a group of healthy young and elderly women. Reliability was assessed using the ICC (2,1). Bartko [2] reported guidelines for ICC values, with ICC values between 0.8 and 1.0 as excellent, 0.6 and 0.8 as good and <0.6 as poor. Our results indicated good to excellent test–retest reliability with the level of reliability being different among different levels of tasks difficulties. The greatest reliability for young women was reported during the test when standing on a compliant surface with eyes closed with six out of eight variables having excellent test–retest reliability followed by standing on firm surface and eyes closed. This agrees with the findings of Bauer et al. [3] reporting higher ICC in the test with eyes closed. While in the elderly group, the test–retest reliability was excellent during the least demanding conditions—firm surface eyes open test. Bauer et al. [4] in a group of elderly community dwelling persons reported slightly better test–retest reliability in more demanding conditions.

In our study, the most reliable parameters in standing on a *firm surface* with eyes open or closed for young women are sway area as calculated with PCA (ICC = 0.77), sway area as calculated with FAO and antero-posterior path length (ICC = 0.85). In the elderly group, mean velocity with ICC = 0.91 (eyes open) and medio-lateral path length ICC = 0.93 (eyes closed) had the highest test–retest reliability. Similar results were reported by Lin et al. [14] with mean velocity being the most reliable variable, followed by the sway area. Their ICC ranged from 0.41 to 0.91. Measurement of mean velocity has most often been reported to be the most reliable CoP variable [7, 14, 15, 23, 24, 28]. The least reliable parameter in both conditions is medio-lateral variability (ICC = 0.56, ICC = 0.40, respectively) in a group of young women, and medio-lateral variability and sway area as calculated with FAO (ICC = 0.80, ICC = 0.61 respectively) in a group of elderly women.

Test–retest reliability of the postural sway while standing on a *compliant surface* with eyes open or closed is being understudied [11, 28]. For these two measurement conditions, the test–retest reliability in our study was higher in the eyes closed condition. In the group of young participants, the most reliable parameter for the test with eyes open is medio-lateral variability (ICC = 0.83). For the test with eyes closed, the most reliable parameters are total path length, mean velocity and medio-lateral path length, all showing excellent reliability (ICC = 0.90). Salavati et al. [28] also reported a good test–retest reliability, especially for mean velocity (ICC = 0.74) in a group of young persons with musculoskeletal problems, while Harringe et al. [11] reported lower degree of repeatability in young injured gymnasts. In the elderly group, mean velocity was the most reliable for both testing conditions (ICC = 0.92, ICC = 0.90, respectively). The results are in agreement with Moghadam et al. [15] who reported good test–retest reliability for mean velocity (ICC = 0.78). The least reliable parameter for eyes open and eyes closed in both groups is antero-posterior variability (ICC = 0.67, 0.66, and ICC = 0.72, 0.63, respectively). The variability in our study is expressed as standard deviation of the position of CoP on the force platform in the time series. This simple measure of variability is used as a CoP variable, and its test–retest repeatability has been also reported as poor [11, 28]. Based on our results and supported by previously reported studies, we can therefore not recommend the use of standard deviation as a reliable outcome measure for balance assessment.

When comparing results of the time-domain variables of mSIT between young and elderly women with eyes open on both types of support surface, higher ICC values were found in elderly group for all variables on firm surface and for seven out of eight variables when standing on a compliant surface. In the eyes closed conditions, the results of both groups indicated similar test–retest reliability. The fractal dimension variables had frequently higher ICC for elderly women on firm surface at eyes open and closed (Tables 2, 3, 4 and 5). These results indicate a better test–retest reliability of mSIT for elderly as compared to young women. In general, higher test–retest reliability is indicated in more demanding sensory conditions as compared to less demanding sensory conditions in a group of young women. These results are in agreement with previous observations where more demanding conditions decreased the normal postural variability, and this could be interpreted as a hallmark of normal balance performance [5]. For the elderly, the results indicated that their postural integration system might already be challenged in a less demanding condition such as standing on a firm surface.

The test–retest reliabilities of the fractal dimensions of postural sway variables were generally lower in both groups of participants as compared to the time-domain variables. The results indicated that fractal dimension is not as reliable method of postural sway analysis as the time-domain ones and could therefore not be recommended for the use as a treatment outcome measure. These results contradict the ones of Doyle et al. [7] who determined high reliability of the fractal dimensions as opposed to the time-domain variables. Their calculation procedure for fractal dimensions was the same as ours with the exception that they performed only 10-s measurements and used only the total time interval, whereas our dimensions were calculated from 60-s measurements and for the short and long time intervals separately. This averaging over the two time intervals could probably hide the differences between measurements and therefore causing higher ICC values.

Test–retest reliability of mSIT as a whole was studied by Harringe et al. [11] in a sample of elite female gymnasts who were divided into 4 groups: uninjured group, low back pain group, lower extremity injury group and multiple injury group. The ICC ranged from 0.31 to 0.66 when standing on a firm and compliant surface with eyes open, and ICC ranged from 0.14 to 0.82 when standing on a firm and compliant surface with eyes closed. Their results also indicated greater reliability when standing with eyes closed, and the variable with the greatest ICC was the path length in both conditions, with eyes open and closed. The higher level of test–retest reliability in more demanding conditions was reported also by Bauer et al. and Bauer et al. [3, 4]; i.e. the most demanding conditions were the most reliable. On the other hand, Moghadam et al. [15] reported higher test–retest reliability in the least demanding conditions. However, the compliant surfaces in the reported studies were not equal; therefore, direct comparison of the results is difficult since the foam density and its elastic modulus have been reported to affect the CoP measurements [17].

Given the increasing number of studies using compliant surface in their training method [10], measurements of postural sway on a compliant surface separately or as part of mSIT had been used as one of the outcome measures [21, 26, 35] for balance-specific exercise programmes for elderly, [9, 30], stroke [13] and sportsman rehabilitation [18]. Our results indicate that mSIT with its four sensory conditions is a reliable measure for evaluating the balance, especially for the elderly, while its minimal clinically significant difference is yet to be defined.

## Conclusion

Modified sensory interaction test measured on force platform has good to excellent test–retest reliability in a group of young and elderly women. Higher degree of test–retest reliability is typical for more demanding conditions in the group of young women. Of the eight evaluated time-domain CoP variables, mean velocity and sway area indicated the highest test–retest reliability, while for the fractal dimensions mSIT indicated poor to good test–retest reliability. The use of mSIT on a force platform with time-domain variables could therefore be recommended as an outcome measure for balance retraining programmes.
